# A Game-Based School Program for Mental Health Literacy and Stigma Regarding Depression (Moving Stories): Protocol for a Randomized Controlled Trial

**DOI:** 10.2196/11255

**Published:** 2019-03-14

**Authors:** Anouk Tuijnman, Marloes Kleinjan, Evert Hoogendoorn, Isabela Granic, Rutger CME Engels

**Affiliations:** 1 Behavioural Science Institute Radboud University Nijmegen Netherlands; 2 Trimbos Institute Utrecht Netherlands; 3 Department of Interdisciplinary Social Sciences Utrecht University Utrecht Netherlands; 4 IJsfontein Amsterdam Netherlands; 5 Erasmus School of Social and Behavioural Sciences Erasmus University Rotterdam Netherlands

**Keywords:** depression, help-seeking behavior, helping behavior, health literacy, stigma, video games, adolescence, secondary schools

## Abstract

**Background:**

The prevalence of elevated depressive symptoms among youth in most western societies is high. Yet, most adolescents who are experiencing depressive symptoms do not seek help. Low mental health literacy, high stigma, and low social support have been shown to hinder help-seeking. A small number of interventions has been developed to target mental health literacy and stigma, but few focus on actual help-seeking and first aid behavior. We have developed a game-based school program called Moving Stories that targets mental health literacy, including knowledge and behavior, and stigma among adolescents, in regard to depression specifically.

**Objective:**

Our aim is to describe the protocol for a study that will test the effectiveness of the program Moving Stories in a Dutch adolescent sample. We hypothesize that adolescents who participate in the program Moving Stories will have better mental health literacy and less stigma regarding depression compared to adolescents in the nonintervention control group at posttest and at 3- and 6-months follow-up. We also expect a positive change in actual help-seeking and first aid behavior at 3- and 6-months follow-up.

**Methods:**

Moving Stories has been developed by a professional game design company in collaboration with researchers and relevant stakeholders. The effectiveness of Moving Stories will be tested through a randomized controlled trial with two conditions: Moving Stories versus control. Participants will fill in questionnaires at pretest, posttest, and 3- and 6-months follow-up. Our power analysis showed a required sample size of 180 adolescents.

**Results:**

Four high schools have agreed to participate with a total of 10 classes. A total of 185 adolescents filled in the pretest questionnaire. The last of the follow-up data was collected in December 2018.

**Conclusions:**

If Moving Stories proves to be effective, it could be implemented as a school-based program to target mental health literacy and stigma regarding depression; this could, in turn, improve early help-seeking in adolescents suffering from depression.

**Trial Registration:**

Nederlands Trial Register NTR7033; https://www.trialregister.nl/trial/6855

**International Registered Report Identifier (IRRID):**

DERR1-10.2196/11255

## Introduction

### Background

Depression is considered to be one of the leading causes of disability worldwide. A recent World Health Organization report indicated that 4.4% of the world population is suffering from a depressive disorder [1]. Although the prevalence of depressive disorders in youth is lower than in adults [1], the numbers of young people experiencing elevated depressive symptoms are substantial. In a recent large-scale European study, approximately 40% of adolescents were suffering from clinical or subclinical depressive symptoms [2], which put them at increased risk for developing a depressive disorder in adulthood [3,4]. Furthermore, in adolescence, both symptoms of depression as well as a full-blown depressive disorder have been related to academic problems [5,6], social problems [5], physical problems, [5] and suicidal ideation [4]. In view of these long-term negative consequences, it seems important that young people seek the help they need to deal with depressive symptoms.

Even though many young people are experiencing depressive symptoms, over half of them do not seek help [7]. Some of the barriers to help-seeking are low mental health literacy, perceived stigma, and a preference for self-reliance [7,8], while social support and encouragement by others to seek help are related to increased help-seeking [7]. It is important to overcome these barriers and encourage social support, as prolonged time between depression onset and actual treatment leads to a diminished response to treatment and a smaller chance of remission [9]. Considering the high prevalence of depressive symptoms, the negative consequences, and the benefits of seeking help early in the process, it is relevant to target the causes that hinder help-seeking behavior in young people. Consequently, we have developed Moving Stories, a game-based school program that targets both mental health literacy and stigma regarding depression in young adolescents.

### Mental Health Literacy and Stigma

Mental health literacy has been defined as “knowledge and beliefs about mental disorders which aid their recognition, management, or prevention” [10]. Mental health literacy does not only refer to knowledge, but also to connected actions [11], not only by those who need help, but, equally important, by the people close to them.

With regard to recognizing a disorder, multiple studies have shown that both adolescents and adults find it difficult to recognize a mental health disorder [11,12]. Compared to adults, adolescents seem to be even less able to identify a disorder like depression [13]. Therefore, especially in adolescents, it is crucial that symptom and disorder recognition improve, as symptom recognition has been linked to choosing appropriate help and treatment [14].

Although increasing people’s ability to recognize a depressive disorder is beneficial for help-seeking behavior, labeling a person as mentally ill has also been linked to stigmatizing attitudes [15]. These attitudes, in turn, have been linked to a diminished amount of appropriate help being offered to peers in need of help [16], possibly counteracting efforts to increase help-seeking. Stigma is also considered to be an important factor as to why people who could benefit from help do not seek help or do not fully participate in treatment [17]. Consequently, this means that in targeting symptom recognition it is also necessary to focus on stigma.

One of the coping strategies that people with mental problems often use is finding social support. Most people view this type of help as potentially beneficial, but it becomes a concern when they turn solely to friends and family instead of seeking professional help [11]. Adolescents prefer seeking help from people they know [18,19] and they believe this help to be beneficial [20]. For adolescents, seeking help only from peers becomes a specific concern, since peers might not be able to provide sufficient or appropriate help [11]. Hence, adolescents could benefit greatly from knowing more about help-seeking options and available treatments. Moreover, teaching adolescents how to help their peers in learning about appropriate first aid for mental health could encourage the seeking of appropriate help among those who need it.

### Mental Health Literacy and Stigma Interventions

Although the research on mental health literacy interventions for youth is scarce, the few existing studies show promising results [12]. One example is of a school-based intervention consisting of short educational sessions in the classroom, involving a trainer who has lived experience with a mental disorder [21]. In a sample of 14-16-year-olds from the United Kingdom and Canada, findings showed an increase in mental health literacy after receiving the intervention. A second school-based intervention with lived-experience trainers targets symptom and disorder recognition with information-delivery sessions, videos, and discussions. This intervention proved to be effective in improving symptom and disorder recognition in Australian adolescents between 14 and 18 years of age [22]. A third school-based mental health literacy program consists of 10 hours of class sessions about mood disorders and helping peers, with teachers providing the program. The program was associated with increased mental health literacy and decreased stigma in Australian 13-16-year-olds [23].

More recently, teen Mental Health First Aid was developed [24]: an adolescent version of the well-studied Mental Health First Aid training for adults [25]. A recent meta-analysis demonstrated that the adult training results in a decrease of negative attitudes and an increase of knowledge and supportive behaviors toward others with mental health problems [26]. For the recent adolescent version, a Delphi consensus study was conducted to find key messages to use in the training to help adolescents provide basic mental health first aid to their peers [27]. Based on those findings, a 5-point action plan was developed that serves as the basis for the training. The first two effect studies showed promising results, with mental health literacy increasing and stigma decreasing in participating adolescents [24,28].

The research on mental health literacy interventions is scarce, but more work has been conducted on the effectiveness of antistigma programs. Most studies on decreasing stigma among youth reveal that education and contact with a person with lived experience—someone who has suffered from a mental health disorder him- or herself—are related to decreases in stigma [29]. A meta-analysis showed that education and contact were equally effective in decreasing stigma [29], while a review showed that personal contact was more important in reducing stigma among young people [30].

Even though there are promising programs for mental health literacy and stigma among youth, there are still few interventions that target actual help-seeking behavior. Most intervention programs focus on enhancing knowledge, but not on enhancing behavioral styles [11]. We have developed a game-based school program called Moving Stories. While most mental health literacy programs consist of didactic sessions, we argue that the nature of video games fits better with our goal of teaching skills alongside increasing knowledge. Already, there are several examples of video games successfully teaching youth health knowledge and skills (eg, in cancer treatment specifically [31] and healthy lifestyles in general [32]) and changing mental health stigma [33]. Games provide the opportunity to practice behavior in a relatively safe, engaging, and virtual environment and to learn by doing [34]. Moreover, players usually receive immediate feedback on their actions in games, encouraging them to continue and learn more [35]. Finally, games are an important part of young people’s lives [36], making a game-based intervention relevant for this population.

### Objectives

The goal of this paper is to describe the protocol for a study that will test the effectiveness of the program Moving Stories in a Dutch adolescent sample by means of a randomized controlled trial. Our first hypothesis is that adolescents who participate in the Moving Stories program will have better mental health literacy and will endorse fewer stigmatizing attitudes regarding depression than adolescents who do not participate, both directly at posttest and at 3- and 6-months follow-up. Second, we expect a change in help-seeking and first aid behavior. At 3- and 6-months follow-up, we expect that adolescents in the Moving Stories group, compared to the control group, will have sought more help if they were experiencing depressive symptoms or provided increased appropriate first aid if they were in contact with a peer who was experiencing depressive symptoms.

## Methods

### Design

**Figure 1:**
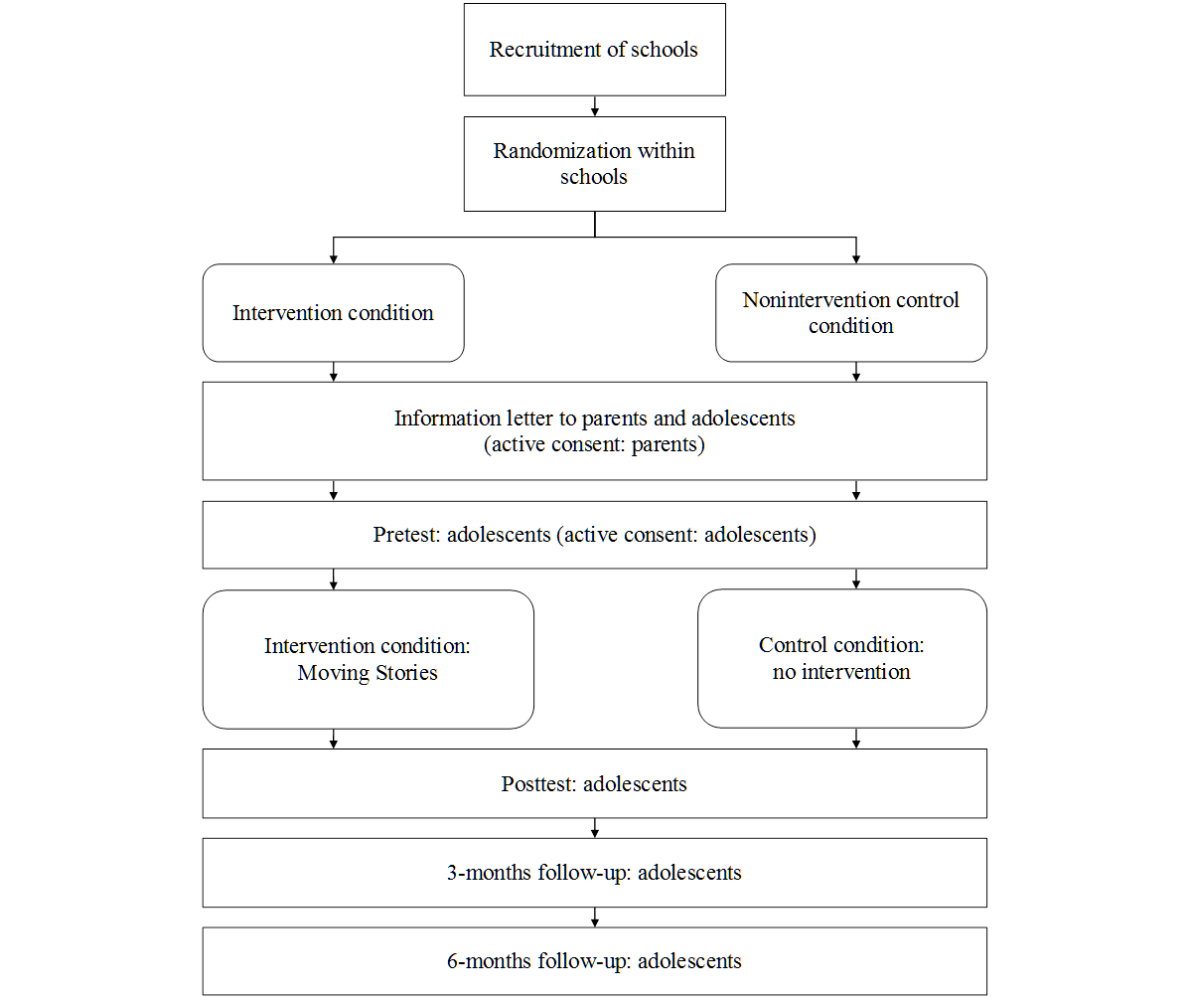
Flowchart of the study design.

The effectiveness of Moving Stories will be tested within a randomized controlled trial with two conditions: Moving Stories versus a nonintervention control group (see Figure 1 for the flowchart of the study design). We have chosen not to include a third condition with an alternative program because there is currently no mental health literacy program for young adolescents available in the Netherlands. Classes within a school will be randomized to either condition to avoid skewed distribution of participants over the two conditions due to school effects. The outcomes will be measured by self-report at four assessment points: T1 (pretest, within one week before the start of the program); T2 (posttest, within two weeks after the end of the program); T3 (3-months follow-up); and T4 (6-months follow-up), with the exception of the behavioral outcomes, which will not be assessed during posttest.

### Program

Moving Stories is a game-based school program, which consists of three parts: (1) an introduction lesson; (2) a single-player, mobile, 3D video game; and (3) a contact session with someone with lived experience with a depressive disorder. The program targets three components of mental health literacy, specifically regarding depression, namely (1) recognition of when a disorder is developing, (2) knowledge of help-seeking options and treatments available, and (3) first aid skills to support others who are developing a mental disorder or are in a mental health crisis [10]. Moreover, the program aims to decrease stigma around depression among youth. Moving Stories has been developed for high school students and should be offered to an entire class. The program is delivered within one week, making it convenient to incorporate into schools.

**Figure 2:**
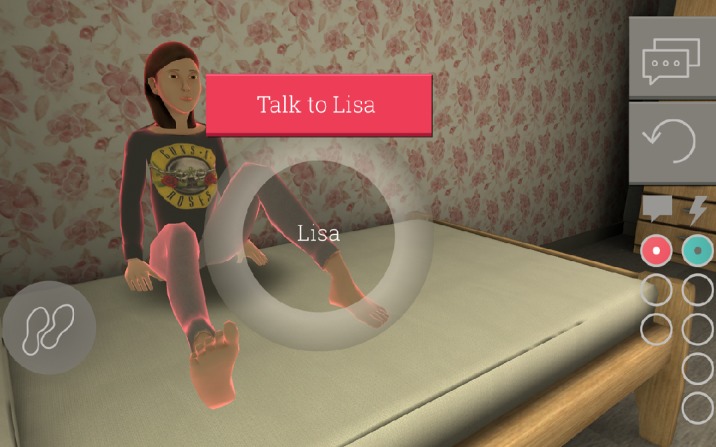
Moving Stories' video game character, Lisa (screenshot; translated to English).

During the introductory lesson, adolescents who were in a class together were asked to download the game on their mobile phone. Moving Stories can be downloaded from the Google Play Store or Apple’s App Store (iOS). All adolescents in the class received a classroom password to be able to play the game. This password was linked to their class schedule, allowing for joint playing time and feedback moments. The adolescents were then able to watch an introductory video about the game. The video game is about a character, Lisa, who is the player’s fictional cousin (see Figure 2). The introductory video talks about who she is and what the relationship is between her and the player. The video also conveys that Lisa has not been feeling well lately. She has lost interest in most things, seems to be somber most of the time, and over the past few days has not gotten out of bed. The player is asked whether they could help her. After the video, the adolescents were told that they would play the game for five days. Each morning, when they would start the game while still at home, they would wake up in Lisa’s house and would be able to do five things for her (eg, get her something to drink). Some of those actions would be positive, while others would be negative. After they would choose five actions, they would go to school and their day would start. During the day at set time points, they would get feedback from Lisa about what they did in the game through automated text messages. They would earn points for their actions; together these points would add up to a total *Relationship* score, which would be shown in the menu of the game. That score would illustrate the quality of their relationship to Lisa and at the same time gives the research team an indication of the adolescents’ first aid skills through the actions they took to help Lisa in a more targeted and constructive way. The adolescents were also told that they could share the messages with each other and that sharing them might give them more information about the game and more quickly improve their skills in the game. Lastly, they were told that if they had questions or wanted to discuss what they had seen in the game before the contact session at the end of the intervention, they could go to their school welfare coordinator or school counselor.

**Figure 3:**
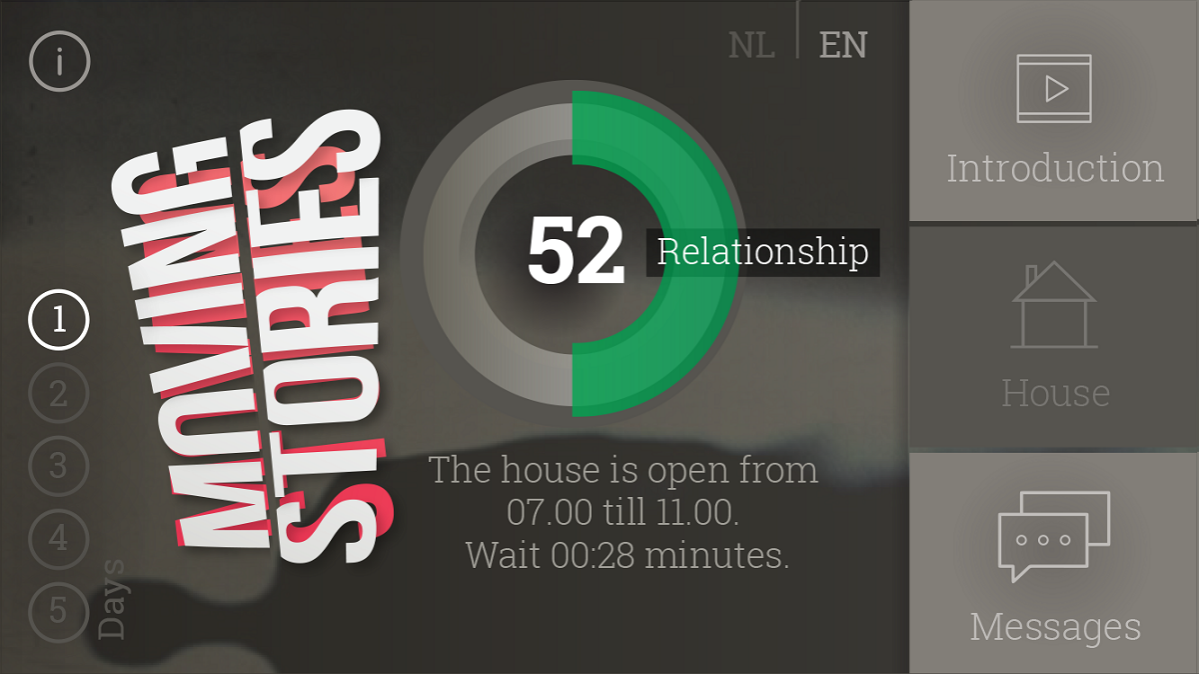
Moving Stories menu (screenshot, translated to English).

The video game has a menu (see Figure 3) with buttons to access the house and Lisa’s messages, as well as a meter for the *Relationship* score. The menu also shows which of the five playing days the player is on and includes a button that leads players to an information page within the game. The information page shows the player’s personal ID and has a button they can press when they “feel like giving up on life.” If the player presses the button they are immediately directed to the 113 Zelfmoordpreventie (Suicide Prevention) website, the Dutch organization for suicide prevention, where they can chat or have a call with a trained volunteer. The 113 Zelfmoordpreventie website helps both adults and youth.

**Figure 4:**
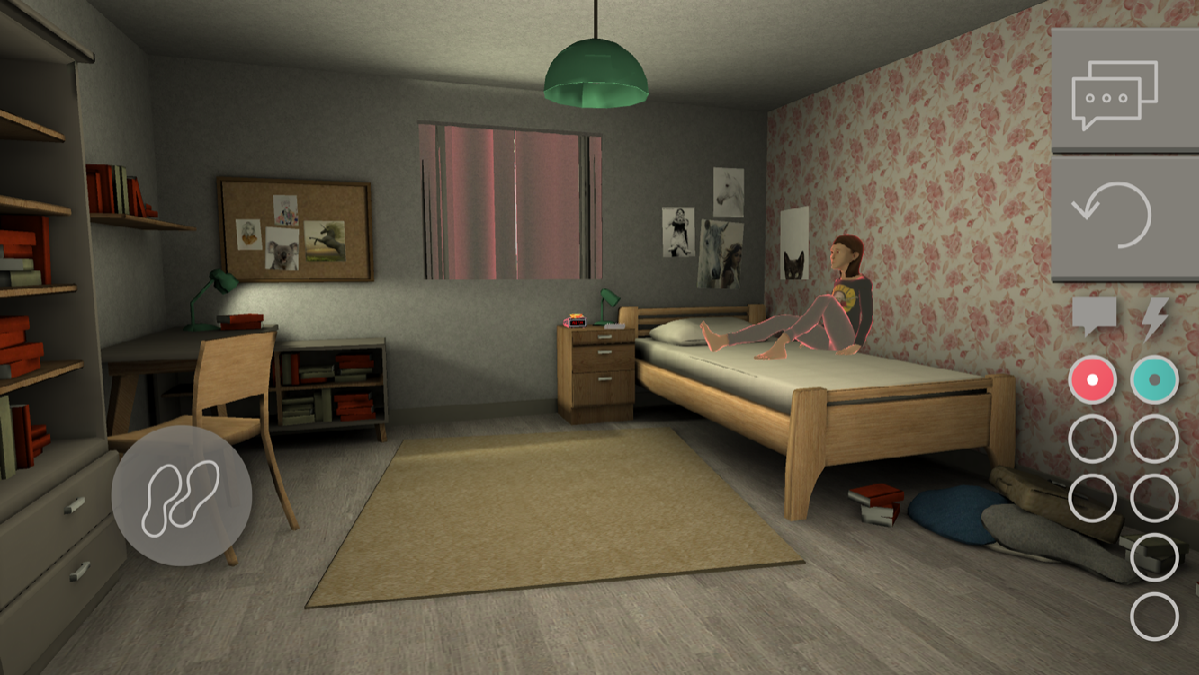
Lisa's house in Moving Stories (screenshot).

Within the house (see Figure 4), players are able to walk around, examine objects, talk to Lisa, and perform actions. Through interactions with Lisa, we aimed to increase the players’ ability to recognize depressive symptoms. There is no limit to walking, examining, or talking; however, players are only able to perform five actions per day, which are all related to good or bad first aid skills [27] and which will increase or decrease the Relationship score with Lisa. Once players perform those five actions, they are asked whether they wanted to go to school or redo the day. Playtime of the game per day is approximately 10-15 minutes. During the day, the entire class receives personal messages at the same time from Lisa, with feedback on their personal actions through text messages. Through the feedback, we aimed to increase players’ knowledge of help-seeking options and improve their first aid skills. The feedback moments are mainly scheduled during break times, so the players will have the opportunity to discuss the messages and talk about game strategies.

The goal of the game is for players to build a relationship with Lisa by showing interest, trying to help her feel a little better, and following up on promises. The end goal of the game is to get an adult involved, after discussing this with Lisa. When the player reaches a *Relationship* score of 50, Lisa gets out of bed. When the player reaches a *Relationship* score of 95, Lisa will be willing to talk to an adult about what is going on. Only when the player discussed this with her, and she mentioned she was willing, does the action of calling an adult get rated positively. After five days of playing, all players see a final scene that takes place a few months into the future. In the scene, Lisa tells the player that she got help and that she is getting better. Based on the final *Relationship* score, Lisa thanks the player for their efforts during the time they were in the house and gives specific feedback on what the player could have done to further help her.

After five days of playing, the program finished with a contact session. In this concluding session, the story of the girl in the game was brought into the real world. A trainer with lived experience with depression led the contact session. All trainers have been trained by the Trimbos Institute in telling their story, guiding a discussion about the game, and translating their story and that of the game to specific first aid skills. In the session, the trainer told their own story and used the experiences of the adolescents in the game to discuss five first aid skills the adolescents could go back to when they want to help a friend who might be suffering from depression. These five skills, or action points, in helping a friend were based on content from the teen Mental Health First Aid training [24] and have been translated into Dutch: (1) Look for warning signs, (2) Ask how they are, (3) Listen without judgment—“without judgement” was added specifically to this program, (4) Help them connect with an adult, and (5) Be a friend. During the contact session, adolescents were allowed to ask questions about the game and about the experience of the trainer. To guide the discussion, the trainer used a PowerPoint presentation with predetermined questions that were related to the content of the game and the five action points. A member of the research team was present to make sure that all questions were discussed.

**Figure 5:**
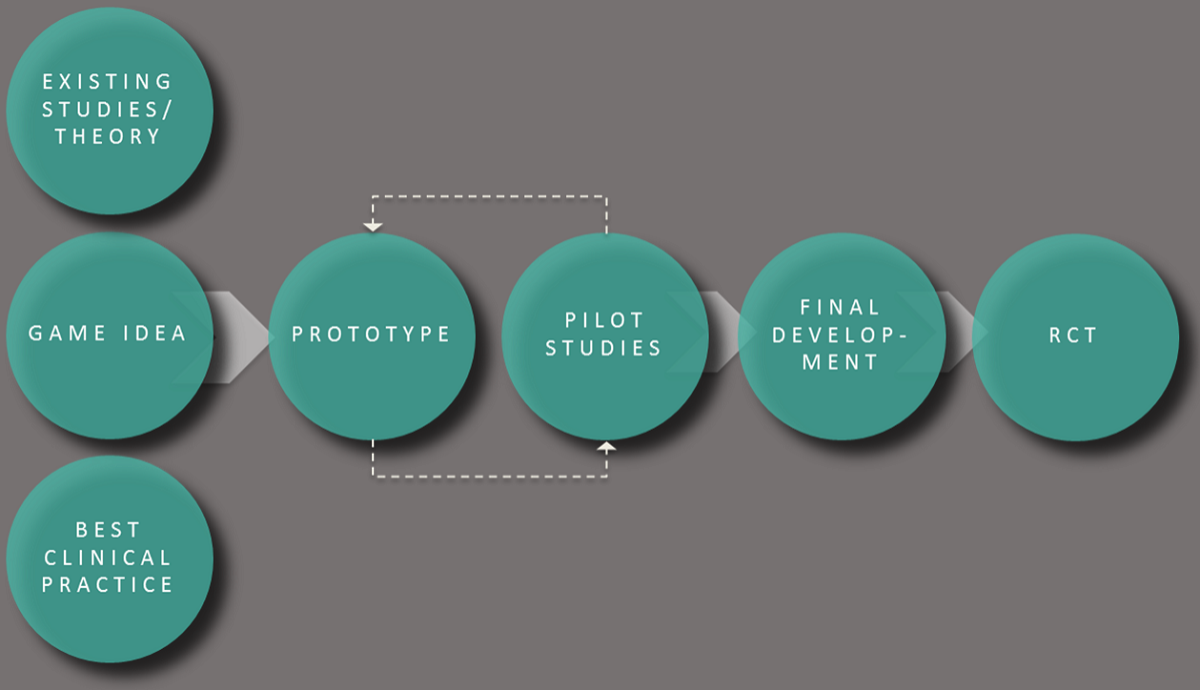
Iterative design process for the development of Moving Stories. RCT: randomized controlled trial.

Moving Stories has been developed through a close collaboration between game designers and behavioral scientists using an iterative design process (see Figure 5). This means that multiple prototypes have been tested in pilot studies before the final version was built. All stakeholder groups have been involved during each development phase, including youth, therapists, and teachers. The concept of the game was based on the latest literature and best practice experiences. In total, we ran through five iteration rounds, each with several playtests. Over 200 people played the game before final development.

### Procedure

Participants are adolescents in the second year of high school (ie, 12-15 years old). Schools in the Netherlands were approached and asked to participate with at least two second-year classes to allow for within-school randomization. The exclusion criterion was refusal from either the parent or the adolescent to participate.

After schools agreed to participate, the classes were allocated to either the Moving Stories or control condition by an independent researcher using computer-generated random numbers. All parents and adolescents in the participating classes received an information letter via the school with information about the study (see Multimedia Appendix 1). Along with the letter, parents received a consent form, which they had to sign and send back to the researchers in order to give consent for their child’s participation. Before adolescents filled in the pretest, they will also had to give written active consent for participation. All questionnaires at all assessment points were Web based and were either filled in at school or at home.

To verify that Moving Stories does not increase depressive symptoms or suicidal ideation in adolescents, the Child Depression Inventory (CDI) [37,38] was administered. At all assessment points, if adolescents had a score on depressive symptoms in the clinical range and/or suicidal intention (CDI>29 and/or a score of 2 on question 9 of the CDI) [37,38], both the parents and adolescent were contacted by phone by a clinically trained member of the research team to inform them of the result and to give advice on where to seek professional help. We follow standard Dutch guidelines, in which we refer adolescents and their parents to the appropriate professional channels of care, where they will receive an assessment and subsequent intake if necessary. No follow-up of this group will be initiated from the research team itself. This procedure was approved by the Research Ethics Committee and complies with Dutch care guidelines. Adolescents who were contacted were not excluded from participating in the study, since that would increase stigma. During analyses, we will check and, if necessary, control for the possible effects of contacting participants and referring them to care.

To make sure that all adolescents who seek help from teachers during the study receive the help that they need, all teachers were provided with an information booklet developed for this study that includes short practical tips on what they can do to help their students. The primary researcher informed the teachers and other professionals involved at the school about the study and the short practical tips at an information session. In addition, teachers had the opportunity to follow an e-learning program on suicidality and depression in youth. This program, called Mental Health Online [39], aims to improve knowledge and self-confidence in people working with youth and has been found to be effective [40]. For this study, the developers of Mental Health Online have added an information page on depression to the program, since the e-learning program mainly discusses suicidality in youth [41] (in Dutch).

At pretest, adolescents were provided with a participant number by the researcher to fill out the questionnaires. They also received a personal player ID in the game. The data from the questionnaire and the data from the game will be matched through the participant number and personal player ID. Adolescents filled in their names and contact information during the pretest; this information can only be accessed by the primary researchers (AT and MK) and data management team at the Trimbos Institute (Dutch National Institute on Mental Health and Addiction) for the purpose of calling adolescents and their parents in case of clinical depressive symptoms and/or suicidal ideation. Adolescents were reminded, through email or via a phone call, to fill in the questionnaires. Data are stored on a secure server in accordance with European privacy law.

We expected that most adolescents would have had a suitable mobile phone or tablet that can run the video game Moving Stories. If an adolescent did not have a mobile phone or tablet that was suitable for the video game, a mobile phone was provided by the research team for the duration of the program. Adolescents received €12.50 in total for filling in the questionnaires at the four assessment points. Both parents and adolescents were allowed to withdraw their consent at any time during the study without consequences. Ethical approval for this study was provided by the Ethical Committee of the Faculty of Social Sciences at the Radboud University Nijmegen (ECSW2017-2306-526). This study is registered in the Dutch Trial Register (NTR7033).

### Measures

#### Descriptives

Sociodemographic variables include gender, age, ethnicity, and current educational level. We measured prior gaming experience by asking participants if they play video games and, if so, on what platforms and how many hours per week they play video games.

#### Depressive Symptoms

The CDI [37,38] was used to control for depressive symptoms. The questionnaire consists of 27 items, each with three statements to choose from. Participants were asked to pick the statement that best described how they felt in the previous two weeks. An example of an item’s three statements is as follows: *I sometimes feel sad; I often feel sad; I always feel sad*. Each statement corresponds to a score of 0, 1, or 2. Scores are added up to a total score for depressive symptoms. The Dutch version of the CDI has good internal consistency and test-retest reliability [42].

#### Outcome Measures

##### Overview

Mental health literacy regarding depression was measured by (1) symptom recognition, (2) first aid intentions, (3) knowledge of first aid, (4) first aid confidence, (5) beliefs about help, and (6) help-seeking intentions. Depression stigma was measured by (1) personal stigma, (2) perceived stigma, and (3) social distance. For an overview of all measures used and the relative assessment points, see Multimedia Appendix 2. All measures that did not have a Dutch version have been translated into Dutch and translated back into English by an independent researcher. Incongruities were discussed and resolved. Cronbach alpha values will be calculated for all measures with more than one item.

##### Symptom Recognition

Symptom recognition was assessed by using three vignettes with gender-matched descriptions of 15-year-old adolescents with depression, social anxiety, and psychosis, respectively [43]. Participants were asked what might be going on with this person. Responses were open ended. The symptom *irritability* has been added to the depression vignette, since this is considered an important symptom in adolescence [44]. Correct scores in the depression vignette are defined by labeling the person as depressed or by using a word related to depression. Overestimation of depression is defined by labeling the person in the social anxiety and/or psychosis vignette as depressed or by using a word related to depression. For the following items, the vignette of the person with depression was used as an example.

##### First Aid Confidence

Confidence in providing first aid was measured by asking how confident the participant would be to help the person in the vignette if he or she was their friend: 5-point scale from 1 (not at all confident) to 5 (very confident) [24].

##### First Aid Intentions

To measure general first aid intentions, participants were asked how much they agree with the statement “If [name] was a friend, I would help him/her”: 5-point scale from 1 (totally disagree) to 5 (totally agree). Specific first aid intentions were assessed by asking whether they would perform the mentioned first aid action “if [name] was a friend”: 5-point scale from 1 (never) to 5 (certainly). In total, six helpful (eg, “Tell [name] I have noticed something seems wrong and I want to make sure s/he is okay”) and six harmful (eg, “Ignore [name] because s/he is being attention-seeking”) actions were mentioned [28] and an open-ended option was provided (eg, “I would do something else other than the options mentioned above, namely...”). The scores for the harmful actions will be reverse scored. A total score for first aid intentions per scale will be calculated by summing up the scores for the helpful actions and the reverse-scored harmful actions, with higher scores representing better first aid intentions. Both scales have acceptable-to-good reliability [28].

##### Beliefs About Help

Beliefs about help were assessed by asking whether the following 10 people would make person’s situation in the vignette *better*, *not better*, *not worse,* or *worse*: (1) boyfriend or girlfriend; (2) friend (not related); (3) parent; (4) other relative; (5) psychologist or social worker (outside school); (6) phone helpline; (7) general practitioner; (8) teacher; (9) school welfare coordinator or school counselor; and (10) religious leader (eg, priest, imam, or rabbi). They were also asked who of those 10 would be most helpful. The number of selected adult sources deemed to be helpful (*better*) will be used to calculate beliefs about appropriate help: the *other relative* category will be excluded, since it could include an adult *or* peer [24].

##### Stigma

Both personal and perceived stigma was measured using the Dutch Depression Stigma Scale [45]. Any mention of the word *depression* was substituted by the situation of the person in the vignette (eg, “[name] is *dangerous*,” similar to the procedure in Jorm and Wright [46]). The last two statements in the scale about hiring a person with depression and voting for them if they were a politician were not used because we do not consider those relevant for this age group. The original personal and perceived stigma scales both have acceptable-to-good internal consistency [47]. Social distance was measured with the five items from the Social Distance Scale for youth [46]. The Social Distance Scale for youth has excellent internal consistency [28].

##### First Aid Behavior

First aid behavior was measured by asking whether the participant has had contact with someone who has experienced a problem similar to that seen in the vignette within the last three months. A problem is defined as when “someone has changed a lot in his/her normal thoughts, feelings, and behavior, which made it hard for him/her to move on with his/her life. The situation did not resolve itself and went on longer than you had expected.” If the participant answered *yes* or *maybe*, they were then asked whether they offered the other person their help. If so, or if they are unsure, they were asked what they did out of the 12 actions and the open-ended option mentioned in the First Aid Intentions section above. First aid behavior will be calculated similar to that of first aid intentions [28].

##### Help-Seeking Intentions

Help-seeking intentions were measured using the General Help-Seeking Questionnaire (GHSQ) [48], to which we added the options *teacher* and *school welfare coordinator/school counselor* to match all sources of help to the items in the Beliefs About Help section. Average scores for the following three categories will be calculated: (1) general help-seeking intentions, (2) help-seeking intentions using informal sources, and (3) help-seeking intentions using formal sources. Higher scores will indicate higher intentions. The GHSQ has good internal consistency and excellent test-retest reliability [48].

##### Help-Seeking Behavior

Help-seeking behavior was assessed by asking whether the participants themselves had experienced a problem similar to the situation in the vignette. If they responded with *yes* or *not sure*, they were asked whether someone has helped them with this problem in the last three months and who this person was: multiple options were allowed. We did not distinguish between whether the help was provided to the participant or whether they sought out the help from this person themselves. The same list of people from the Beliefs About Help section and the GHSQ [24,48] was used with the additional response option *Don’t know for sure*. If the participant indicated their boyfriend or girlfriend and/or a friend had helped them, they were asked what that person did out of the 12 first aid actions and the open-ended option mentioned in the First Aid Intentions section above [28]. This was done in order to assess the first aid skills in youth surrounding the person who had experienced a problem such as that in the vignette.

##### Evaluation of Moving Stories

During posttest, participants in the intervention group were asked to evaluate the program Moving Stories across seven items using a 5-point scale from 1 (totally disagree) to 5 (totally agree); for example, “How much do you agree with the following statement? I would like to play the game another time to get a better score.” To distinguish between the different components of the program and the study (ie, game, evaluation session, and research), the participants were also asked which of the components they would recommend to a friend if they had the opportunity to participate in the study over the next year.

##### Contamination Check

To check for possible contamination effects due to the within-school randomization, the participants in the control group were asked at 6-months follow-up whether they had heard of the game Moving Stories and, if so, whether they had played it. If they indicated they had played it, they were asked on what platform they did so.

### Sample Size

The sample size was based on the expected difference (Cohen *d*=0.40) between the intervention and control condition for mental health literacy and stigma at 3-months follow-up, based on Perry et al [23]. We performed a power analysis using Stata version 14.2 (StataCorp) [49], assuming baseline-adjusted regression analyses (alpha=.05; beta=.20). Our provisional estimates for the correlations between pre- and posttest and between posttest and 3-months follow-up are .50. A coefficient of variation of .19 (estimated mean cluster size=18; estimated cluster size range=11-25 [50]) and an intracluster correlation coefficient of .02 [23] lead to a design effect of 1.35. Taking into account the design effect, we calculated that we need 3.75 classes per condition to show the expected effect, rounding up to four classes per condition, with 18 adolescents per class. To adjust for a *t* distribution [51], we added one class per condition, resulting in five classes (ie, 90 adolescents) per condition and a total necessary sample size of 180. Since we will perform the analyses according to the intention-to-treat principle, dropout was not taken into account.

### Statistical Analyses

Descriptive statistics will be calculated for all variables of interest (eg, knowledge of first aid, stigma, and help-seeking intentions). In order to assess whether randomization results in similar groups, we will examine whether there are differences between the two conditions on relevant covariates (ie, sex, age, educational level, ethnicity, gaming behavior, and depressive symptoms) using *t* tests for the continuous variables and chi-square tests for the categorical variables. Variables that are distributed differently between the two conditions will be entered as control variables in all models testing the effects of the conditions.

We will perform our analyses according to the intention-to-treat principle, meaning we will include all children who filled in the pretest questionnaires in the analyses to test the study hypotheses. We will control for clustered data because children are nested within classes. We will use Mplus version 8 (Muthén & Muthén ) [52] for the analyses, since it has special features to deal with missing data. It also allows for analyzing complex data while controlling for clustering. To test whether children in the experimental condition have shown an increase in mental health literacy and a decrease in stigma at 3- and 6-months follow-up, compared to the control condition, regression analyses will be conducted. Both the effect sizes and the confidence intervals will be reported. Open-ended questions will be analyzed exploratively and indicatively where necessary.

## Results

As of the writing of this paper, four schools and 10 classes have agreed to participate in this study. At pretest, 185 adolescents and their parents gave consent and filled in the first questionnaires. The last of the follow-up data was collected in December 2018.

## Discussion

This paper describes the protocol of the first study that will be used to test the effectiveness of the mental health literacy program Moving Stories, which not only targets knowledge and beliefs, but also aims at training youth in both help-seeking and first aid behavior, specifically regarding depression in youth. This project is unique in its use of an online game to teach mental health literacy. Moreover, the program has been designed by professional game designers in close collaboration with relevant stakeholders. We used a rigorous experimental design to answer our research questions. Our study adds to the latest literature on mental health literacy, as there is a lack of intervention studies in adolescents.

Since we randomized within schools and between classes, there is a risk of contamination between the classes. However, considering the necessary sample size, randomizing between schools would potentially lead to large school differences and we have estimated the between-class risk of contamination to be lower than the between-school effect. We will check for potential contamination effects at the last measurement point. Another limitation is that we were unable to objectively assess what the adolescents discussed outside the game. We only measured what they did within the game and assessed outcomes through self-report questionnaires; therefore, we will be unable to say anything about the possible effect of these discussions. Lastly, although we asked about provided first aid behavior, we did not include any objective behavioral measures in this study. Conclusions about actual first aid behavior are therefore limited.

If Moving Stories proves to be effective, it could be implemented as a school-based program to target mental health literacy and stigma and, in turn, improve early help-seeking. Combining the program with screening questionnaires on depression could further aid early detection of mental health problems in adolescents.

## Appendix

Multimedia Appendix 1 Information letters for parents and adolescents (translated to English).

Multimedia Appendix 2 Measured variables and respective assessment points.

## Abbreviations

CDI: Child Depression Inventory
GHSQ: General Help-Seeking Questionnaire
NWO: Netherlands Organisation for Scientific Research
RCT: randomized controlled trial
SMS: short message service
T1: pretest
T2: posttest
T3: 3-months follow-up
T4: 6-months follow-up
