# Supplementary material for: A Game-Based School Program for Mental Health Literacy and Stigma Regarding Depression (Moving Stories): Protocol for a Randomized Controlled Trial
Source: JMIR Res Protoc. 2019 Mar 14;8(3):e11255. doi: 10.2196/11255 (PMC6437615; doi:10.2196/11255)
Supplement: Multimedia Appendix 1 [file resprot_v8i3e11255_app1.pdf]

Dear,

Your school, together with the Trimbos Institute, is paying extra attention to the social and emotional development of youth. For this reason the Trimbos Institute, together with the Radboud University Nijmegen and a game design studio in Amsterdam, has developed a new app. The app consists of a game you can play. In the school year 2017/2018 the Trimbos Institute and the Radboud University Nijmegen will research this app at your school.

What is the app about?

Your entire class will play the game on your own smartphone in week [..]. This means that you will play the game for about 15 minutes in the morning for 5 days. You play the game alone, but your entire class will be playing on the same days. During the day you will also get feedback about the game you've played in the morning.

In the game you are going to take care of a girl. This girl is a virtual character and does not exist in real life. What you do in the app will be processed confidentially.

After playing?

After playing the game you will discuss your experiences with the game with your entire class in a mentor hour. A trainer from the project and a researcher from the research team will guide the discussion. Your mentor will be present too. You will get more information and you will get the opportunity to share your experiences with the game with others.

What does the study entail?

To study what you think of the game and what you've learned from the game and the discussion afterwards, we ask you to answer questionnaires on 4 time points (before and after playing the game and after 3 and 6 months). Besides questions about what you think of the game and what you have learned, we will also ask you for a few personal details (your name, date of birth, mother country, ethnic group, phone number and email address). We ask for your name, date of birth, mother country and ethnic group to be able to execute the study. We need your email address and phone number to be able to remind you to fill in the questionnaires. Your personal details will be deleted 5 years after this study has been finished. All information will be treated confidentially by the Trimbos Institute. The personal details will be stored securely and not shared with others (this also includes your school). If people want access to general information for scientific purposes they can request access with the main researcher. In that case they will only be able to see anonymous data, so they will not be able to see that you have participated in this study, or what your answers were.

In exceptional cases the data can show that you are not feeling well at the moment. In those cases you and your parent(s) or guardian can be informed about this by the researcher. If you do not wish to be informed about this, or if you do not want your parent(s) or guardian to be informed, you cannot participate in the study.

In case you don't give permission for participating in the study, you can't join the program in the class. You will do something else at school during those times. For the time you have spent on filling in the questionnaires you get 12,5 euros in gift certificates.

How do you give permission?

If you want to participate in this study, you can fill in a consent form when you fill in the first questionnaires. You will fill in the questionnaires on [date]. Your parent(s) or guardian will also get an information letter and will be asked to give consent for your participation too. You and/or your parent(s) or guardian can retract your consent for this study at any moment. This has no consequences for you or your parent(s) or guardian. Your information will be deleted from the study.

More information?
If you have a question about this project, you can contact Anouk Tuijnman (a.tuijnman@pwo.ru.nl).

With best regards from the entire project team of the Trimbos Institute and the Radboud University Nijmegen,

Anouk Tuijnman, MSc
Prof. dr. Rutger Engels
Prof. dr. Marloes Kleinjan
Prof. dr. Isabela Granic

Trimbos instituut Behavioural Science Institute Radboud Universiteit

Dear,

Your school, together with the Trimbos Institute, is spending extra attention on the social and emotional development of youth. In the school year 2017/2018 we will conduct a study about that together with the Radboud University Nijmegen.

What is the study about?
The Trimbos Institute has developed a program that concerns the emotional development and social relationships of youth. To test the effect of this program a few classes will participate in the program. Other classes will only fill in questionnaires, so that we can compare the students in the different groups. This is an important aspect of the study, because we want to say something about what students learn from the program.

You are part of the group that only has to fill in the questionnaires.

What does this mean?
All students in your class will be asked to fill in questionnaires about social and emotional development at 4 time points. These 4 time points have been divided over the entire school year. Besides questions about social and emotional topics, we will also ask you for a few personal details (your name, date of birth, mother country, ethnic group, phone number and email address). We ask for your name, date of birth, mother country and ethnic group to be able to execute the study. We need your email address and phone number to be able to remind you to fill in the questionnaires. Your personal details will be deleted 5 years after this study has been finished. All information will be treated confidentially by the Trimbos Institute. The personal details will be stored securely and not shared with others (this also includes your school). If people want access to general information for scientific purposes they can request access with the main researcher. In that case they will only be able to see anonymous data, so they will not be able to see that you have participated in this study, or what your answers were.

In exceptional cases the data can show that you are not feeling well at the moment. In those cases you and your parent(s) or guardian can be informed about this by the researcher. If you do not wish to be informed about this, or if you do not want your parent(s) or guardian to be informed, you cannot participate in the study.

For the time you have spent on filling in the questionnaires you get 12,5 euros in gift certificates.

How do you give permission?
If you want to participate in this study, you can fill in a consent form when you fill in the first questionnaires. You will fill in the questionnaires on [date]. Your parent(s) or guardian will also get an information letter and will be asked to give consent for your participation too. You and/or your parent(s) or guardian can retract your consent for this study at any moment. This has no consequences for you or your parent(s) or guardian. Your information will be deleted from the study.

More information?
If you have a question about this project, you can contact Anouk Tuijnman (a.tuijnman@pwo.ru.nl).

With best regards from the entire project team of the Trimbos Institute and the Radboud University Nijmegen,

Anouk Tuijnman, MSc
Prof. dr. Rutger Engels
Prof. dr. Marloes Kleinjan
Prof. dr. Isabela Granic

Trimbos instituut   Behavioural Science Institute   Radboud Universiteit

Dear sir/madam,

In collaboration with the Trimbos Institute, the school of your child is paying extra attention to the social-emotional development of youth. Young people face many challenges: determining their status within a class, hormonal and physical changes, changes within friendships, a growing need for autonomy, the importance of school achievements, etc. These big and small challenges are not always easy for youth in their daily lives. To do this the Trimbos Institute, together with the Radboud University Nijmegen and a game design studio in Amsterdam, has developed a new app. In the school year 2017/2018 the Trimbos Institute and the Radboud University Nijmegen will research this app at your child's school.

What does this mean for your child?

To give students more insight in social-emotional development and social relationships the class of your child will play a game with the entire class on their smartphone in week [..]. This means that they will play the game for approximately 15 minutes for 5 days (between [..] and [..]). Your child will play the game individually, but the entire class will be playing on the same days. During the day they will get feedback about the game they've played that morning.

What is the content of the app?

The students are supposed to take care of a girl in the game. This girl is a virtual character and does not exist in real life. We hope that by playing the game the students will learn when their friend might not be feeling so well and what they can do for this friend. The goal is that students are more aware of their own wellbeing and that of other students. Data about what the students do in the game will be processed confidentially.

After playing?

After playing the game the students will have a discussion session in the mentor hour at school. A trainer from the project and a researcher from the research team will guide the discussion. The mentor will be present too. They will provide more information and the students will have the opportunity to share their experiences with the game.

What does the study entail?

To study what students learn from playing the game and discussing their experiences with the game, we ask the students who are participating to fill in questionnaire at 4 time points (before and after playing the game and after 3 and 6 months). These questionnaires contain questions, among other things, on game behavior, depressive symptoms, help-seeking skills and stigma. Besides questions about what they think of the game and what they have learned, we will also ask for a few personal details (name, date of birth, mother country, ethnic group, phone number and email address). We ask for their name, date of birth, mother country and ethnic group to be able to execute the study. We need their email address and phone number to be able to remind your child to fill in the questionnaires. All information will be treated confidentially by the Trimbos Institute. The personal details will be deleted 5 years after this study has been finished. The personal details will be stored securely and not shared with others (this also includes the school). If people want access to general information for scientific purposes they can request access with the main researcher. In that case they will only be able to see anonymous data, so they will not be able to trace the data to your child.

In exceptional cases the data can show that your child is not feeling well at the moment. In those cases your child and you as parent(s) or guardian can be informed about this by the researcher. If you or your child does not wish to be informed about this your child cannot participate in the study. It is important to know that the data cannot be interpreted from a psychological perspective. The participation of your child in this study cannot be seen as a psychological test.

In case you don't give permission for the participation of your child in the study, your child can't join the program in the class. They will do something else at school during those times. For the time your child spends on filling in the questionnaires they get 12,5 euros in gift certificates.

This study has been approved by the ethical committee of the Faculty of Social Sciences at the Radboud University Nijmegen.

How do you give permission?

If you want to give consent for the participation of your child in this study, you can fill in the consent form provided with this letter. You can use the provided envelop to send it back. You can do this before [date]. You and your child can decide at any time that you want to stop the participation of your child in this study. Your child's information will be deleted from the study, without this having any negative consequences for you or your child.

More information?

If you have a question about this project, you can contact Anouk Tuijnman (a.tuijnman@pwo.ru.nl).

With best regards from the entire project team of the Trimbos Institute and the Radboud University Nijmegen,

Anouk Tuijnman, MSc
Prof. dr. Rutger Engels
Prof. dr. Marloes Kleinjan
Prof. dr. Isabela Granic

Trimbos instituut   Behavioural Science Institute   Radboud Universiteit

Dear sir/madam,

In collaboration with the Trimbos Institute, the school of your child is paying extra attention to the social-emotional development of youth. Young people face many challenges: determining their status within a class, hormonal and physical changes, changes within friendships, a growing need for autonomy, the importance of school achievements, etc. These big and small challenges are not always easy for youth in their daily lives. In the school year 2017/2018 the Trimbos Institute, together with the Radboud University Nijmegen, will research this topic at your child's school.

What is the study about?

The Trimbos Institute has developed a program in collaboration with the Radboud University Nijmegen that deals with the emotional development and social relationships of youth. To test the effect of this program a few classes will participate in this program. Other classes will only fill in questionnaires, so that we can compare the students in the different groups with each other. This is an important expect of this study, because we want to be able to say whether students really learn something from this program.

Your child is in a class that only needs to fill in the questionnaires.

What does this mean for your child?

All students in the class of your child will be asked to fill in questionnaires at four time points with questions, among other things, on game behavior, depressive symptoms, help-seeking skills and stigma. These 4 time points are divided over the entire school year. Besides questions on social and emotional topics, we will also ask for a few personal details (name, date of birth, mother country, ethnic group, phone number and email address). We ask for their name, date of birth, mother country and ethnic group to be able to execute the study. We need their email address and phone number to be able to remind your child to fill in the questionnaires. All information will be treated confidentially by the Trimbos Institute. The personal details will be deleted 5 years after this study has been finished. The personal details will be stored securely and not shared with others (this also includes the school). If people want access to general information for scientific purposes they can request access with the main researcher. In that case they will only be able to see anonymous data, so they will not be able to trace the data to your child.

For the time your child spends on filling in the questionnaires they get 12,5 euros in gift certificates.

In exceptional cases the data can show that your child is not feeling well at the moment. In those cases your child and you as parent(s) or guardian can be informed about this by the researcher. If you or your child does not wish to be informed about this your child cannot participate in the study. It is important to know that the data cannot be interpreted from a psychological perspective. The participation of your child in this study cannot be seen as a psychological test.

This study has been approved by the ethical committee of the Faculty of Social Sciences at the Radboud University Nijmegen.

How do you give permission?

If you want to give consent for the participation of your child in this study, you can fill in the consent form provided with this letter. You can use the provided envelop to send it back. You can do this before [date]. You and your child can decide at any time that you want to stop the participation of your child in this study. Your child's information will be deleted from the study, without this having any negative consequences for you or your child.

More information?
If you have a question about this project, you can contact Anouk Tuijnman (a.tuijnman@pwo.ru.nl).

With best regards from the entire project team of the Trimbos Institute and the Radboud University Nijmegen,

Anouk Tuijnman, MSc
Prof. dr. Rutger Engels
Prof. dr. Marloes Kleinjan
Prof. dr. Isabela Granic

Trimbos instituut Behavioural Science Institute Radboud Universiteit
