# Supplementary material for: A Game-Based School Program for Mental Health Literacy and Stigma Regarding Depression (Moving Stories): Protocol for a Randomized Controlled Trial
Source: JMIR Res Protoc. 2019 Mar 14;8(3):e11255. doi: 10.2196/11255 (PMC6437615; doi:10.2196/11255)
Supplement: Multimedia Appendix 2 [file resprot_v8i3e11255_app2.pdf]

Appendix 2: Measured variables and respective assessment points.

		Assessment Points			
Concept	Variables	Pre-test	Post-test	3-months FU	6-months FU
**Descriptives**					
	Socio-demographics	X			
	Gaming experience	X			
**Depressive symptoms**					
	Depressive symptoms	X	X	X	X
**Evaluation Moving Stories**					
	Evaluation Moving Stories		X		

Table 1. Measured descriptive and control variables with respective assessment points.

		Assessment Points			
Concept	Variables	Pre-test	Post-test	3-months FU	6-months FU
**Mental health literacy**					
	Symptom recognition	X	X	X	X
	First aid confidence	X	X	X	X
	First aid intentions	X	X	X	X
	Beliefs about help	X	X	X	X
	Help-seeking intentions	X	X	X	X
**Stigma**					
	Personal stigma	X	X	X	X
	Perceived stigma	X	X	X	X
	Social Distance	X	X	X	X
**First aid behavior**					
	First aid behavior	X		X	X
**Help-seeking behavior**					
	Help-seeking behavior	X		X	X

Table 2. Measured outcome variables with respective assessment points.
